# What Is the Ideal Blood Pressure Threshold for the Prevention of Atrial Fibrillation in Elderly General Population?

**DOI:** 10.3390/jcm9092988

**Published:** 2020-09-16

**Authors:** Yoon Jung Park, Pil-Sung Yang, Hee Tae Yu, Tae-Hoon Kim, Eunsun Jang, Jae-Sun Uhm, Hui-Nam Pak, Moon-Hyoung Lee, Gregory Y.H. Lip, Boyoung Joung

**Affiliations:** 1 Division of Cardiology, Department of Internal Medicine, Severance Cardiovascular Hospital, Yonsei University College of Medicine, Seoul 03722, Korea; PYJ221@yuhs.ac (Y.J.P.); HEETYU@yuhs.ac (H.T.Y.); THKIMCARDIO@yuhs.ac (T.-H.K.); SUNNY_JES@yuhs.ac (E.J.); JASON@yuhs.ac (J.-S.U.); HNPAK@yuhs.ac (H.-N.P.); MHLEE@yuhs.ac (M.-H.L.); 2 Department of Cardiology, CHA Bundang Medical Centre, CHA University, Seongnam 13496, Korea; psyang01@cha.ac.kr; 3 Liverpool Centre for Cardiovascular Science, University of Liverpool and Liverpool Heart & Chest Hospital, Liverpool L14 3PE, UK

**Keywords:** atrial fibrillation, hypertension, elderly, prevention

## Abstract

Intensive blood pressure (BP) lowering in patients with hypertension at increased risk of cardiovascular disease has been associated with a lowered risk of incident atrial fibrillation (AF). It is uncertain whether maintaining the optimal BP levels can prevent AF in the general elderly population. We included 115,866 participants without AF in the Korea National Health Insurance Service-Senior (≥60 years) cohort from 2002 to 2013. We compared the influence of BP on the occurrence of new-onset AF between octogenarians (≥80 years) and non-octogenarians (<80 years) subjects. With up to 6.7 ± 1.7 years of follow-up, 4393 incident AF cases occurred. After multivariable adjustment for potentially confounding clinical covariates, the risk of AF in non-octogenarians was significantly higher in subjects with BP levels of <120/<80 and ≥140/90 mm Hg, with hazard ratios of 1.15 (95% confidence interval (CI), 1.03–1.28; *p* < 0.001) and 1.14 (95% CI, 1.04–1.26; *p* < 0.001), compared to the optimal BP levels (120–129/<80 mm Hg). In octogenarians, the optimal BP range was 130–139/80–89 mm Hg, higher than in non-octogenarians. A U-shaped relationship for the development of incident AF was evident in non-octogenarians, and BP levels of 120–129/<80 mm Hg were associated the lowest risk of incident AF. Compared to non-octogenarians, the lowest risk of AF was associated with higher BP levels of 130–139/80–89 mm Hg amongst octogenarians.

## 1. Introduction

Hypertension is the most common comorbidity in patients with atrial fibrillation (AF) and is highly prevalent in patients with AF, especially those aged over 60 years [1]. Elevated blood pressure (BP) is associated with a greater burden of AF [2] and every 20 mm Hg increase in systolic blood pressure (SBP) has a 21% higher risk of AF [3]. The high incidence of hypertension with AF has prompted the argument that AF is another sign of hypertensive target organ damage [4,5,6].

Several epidemiological studies have shown that the levels of SBP 130–139 mm Hg are also associated with increased risk of AF, compared to normal SBP (<120 mm Hg) [7,8,9,10]. Several randomized controlled trials also showed the relationship between BP and risk of AF. In the Cardio-Sis trial (Controllo della Pressione Arteriosa Sistolica trial), the risk of new-onset AF was reduced in the tight control group (SBP < 130 mm Hg) compared to the usual control group (SBP < 140 mm Hg) in patients with hypertension without diabetes [11]. Other report have shown that the intensive therapy group (target SBP < 120 mm Hg) did not show statistical significance with respect to the incidence of AF in patients with hypertension and diabetes [12]. A recent study, using data from Systolic Blood Pressure Intervention Trial (SPRINT) found that intensive treatment with a target SBP of <120 mm Hg in patients with hypertension at high risk of cardiovascular disease reduced the risk of AF [13]. However, the relationship of BP and incident AF has not been established in older subjects.

However, strict BP control can induce serious adverse events such as hypotension, syncope, electrolyte imbalance, and acute kidney injury [14]. The Elderly population is more likely have other risk factors and target organ damage that may be worsened by lowering BP than the younger population [15]. Exacerbation of postural hypotension could be associated with injurious falls, and a low BP targets could be related to an increased risk of reduced renal function amongst octogenarians (age > 80 years) [16]. Of note, the 2018 European Society of Cardiology (ESC) and the European Society of Hypertension (ESH) guidelines recommend less strict BP control for the elderly (age ≥ 65 years) and close monitoring of adverse effects. Additionally, a previous study suggests that intensive BP control had no more benefit than harm in patients with a 10 year-cardiovascular risk of <18.2% [17].

It remains uncertain whether intensive BP lowering to a target SBP of <120 mm Hg results in further lowering of the risk of new-onset AF in octogenarians with hypertension. In this study using the nationwide population-based National Health Insurance Service (NHIS)-senior cohort (NHIS-Senior), we aimed to investigate the optimal BP levels for the prevention of incident AF, defining the ideal BP threshold for the prevention of AF in the general elderly population. Second, we evaluated whether these associations were observed in different age groups and were influenced by strict BP control.

## 2. Experimental Section

Data were collected from the NHIS-Senior, which included about 558,147 individuals, accounting for approximately 10% of the total elderly population over 60 years old in South Korea (approximately 5.1 million) in 2002 [18]. The NHIS-Senior database included the following parameters: sociodemographic and socioeconomic information, insurance status, health checkup examinations, and records of patients’ medical and dental history. These parameters have been stratified to cover 12 years (2002–2013) and anonymized in the cohort study to protect the privacy of individuals. This study was approved by the Institutional Review Board of Yonsei University Health System (4-2016-0179). Informed consent was waived. The NHIS-Senior database used in this study (NHIS-2016-2-171) was made by the NHIS of Korea. The authors declare no conflict of interest with the NHIS.

### 2.1. BP Measurement

BP measurements were obtained at local hospitals and clinics certified for medical health examination centers by the Korean National Health Insurance Corporation. After the patient rested for 5 min in the sitting position, brachial BP was measured by qualified medical personnel at each health examination center. A blood pressure (BP) measurement was repeated if the first measurement was >120/80 mm Hg. Automatic oscillometric devices and mercury sphygmomanometers were used for BP measurements, with the choice of device being at the discretion of individual examination centers. The preferred recommendation stipulated the use of mercury sphygmomanometers until 2015, when the sale of mercury sphygmomanometers was banned. The average of the BP measured at the first and second medical examinations was used for analysis.

### 2.2. Study Population

**Figure 1:**
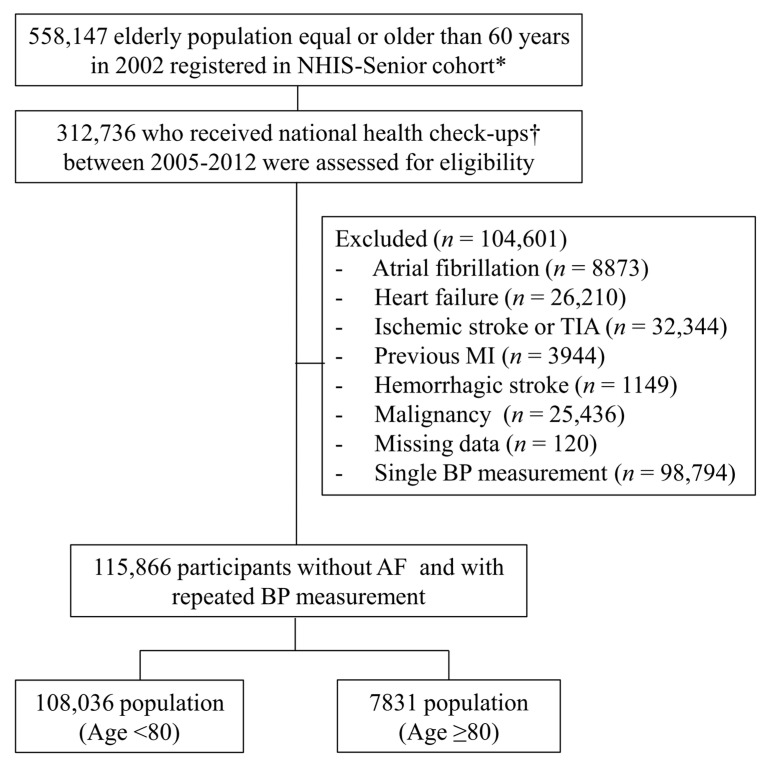
Flowchart of the study population enrollment and analyses. NHIS, National Health Insurance Service; TIA, transient ischemic attack; MI, myocardial infarction; BP, blood pressure; AF, atrial fibrillation. * Korean National Health Insurance Service (NHIS)-Senior cohort. † Complete checkup includes smoking, physical activity, alcohol, BMI, Total cholesterol, blood pressure, fasting glucose.

From the Korean NHIS-Senior, a total of 312,736 patients who had a health checkup between 2005 and 2012 were enrolled, and follow-up data were reviewed until December 2013. The exclusion criteria were as follows: (i) patients who had AF before enrollment (*n* = 8873); (ii) those who had heart failure (HF) before enrollment (*n* = 26,210); (iii) those who had ischemic stroke or transient ischemic attack before enrollment (*n* = 32,344); (iv) those who had myocardial infarction (MI) before enrollment (*n* = 3944); (v) those who had hemorrhagic stroke before enrollment (*n* = 1149); (vi) those who had malignancy before enrollment (*n* = 25,436); (vii) those who had missing data (*n* = 120); and (viii) those who check BP once (*n* = 98,794). Finally, we included 115,866 patients with repeated BP measurement (Figure 1).

### 2.3. Covariates

We obtained information on selected comorbidities in inpatient and outpatient hospital diagnoses. Baseline comorbidities were defined using the medical claims and information about prescription medication prior to the index date. To ensure the accuracy of diagnosis, the patients were considered to have comorbid condition when the condition was a discharge diagnosis or confirmed at least twice in an outpatient setting according to previous studies using the NHIS (Supplementary Materials Table S1) [19,20]. For the status of standard income, the total amount of national health insurance premiums paid by the insured in the year was evaluated in proportion to personal income.

### 2.4. Hypertension and Atrial Fibrillation

Hypertension was defined as the combination of previous hypertension diagnosis (International Classification of Disease-10th Revision (ICD-10) codes) and use of one or more antihypertensive drugs. The hypertension onset date for duration calculations was determined using information on the first date of hypertension diagnosis. The BP status was divided into four groups: (i) SBP of <120 mm Hg and diastolic blood pressure (DBP) of <80 mm Hg; (ii) SBP of 120–129 mm Hg and DBP of <80 mm Hg; (iii) SBP of 130–139 mm Hg or DBP of 80–90 mm Hg; and (iv) SBP of ≥140 mm Hg or DBP of ≥90 mm Hg. The study also compared the SBP status and incidence of AF. Furthermore, the relationship between the DBP status and incidence of AF was analyzed.

AF was diagnosed using the ICD-10, code I48. To ensure diagnostic accuracy, the patients were defined as having AF only when it was a discharge diagnosis or had been confirmed at least twice in the outpatient department. This AF diagnosis definition has been previously validated in the NHIS database with a positive predictive value of 94.1% [19,21].

### 2.5. Statistical Analysis

The baseline characteristics of participants with age over and under 80 years were compared using Student’s *t*-test and Pearson’s chi-square test. The incidence rates of events were calculated by dividing the number of events by person-times at risk, with the 95% confidence intervals (CI) estimated by exact Poisson distributions. Cox proportional hazards regressions were used to compare the incidence of AF with BP status. Two-sided *p*-values <0.05 were considered statistically significant. Statistical analyses were conducted using Statistical Package for Social Sciences (SPSS) version 23.0 (Chicago, IL, USA) and R version 3.3.2 (The R Foundation, www.R-project.org).

## 3. Results

### 3.1. Baseline Characteristics

Compared with non-octogenarians, the octogenarians were predominantly female and had more comorbidities, including hypertension, chronic kidney disease (CKD), anemia, chronic obstructive pulmonary disease, and osteoporosis (Table 1). The low rates of CKD and diabetes for ages in this study might be related with the rigid exclusion criteria of this study. The comparisons of baseline characteristics among patients with different BP levels in non-octogenarians and octogenarians are presented in Supplementary Materials Table S2.

### 3.2. BP and Incident AF in Different Age Groups

**Figure 2:**
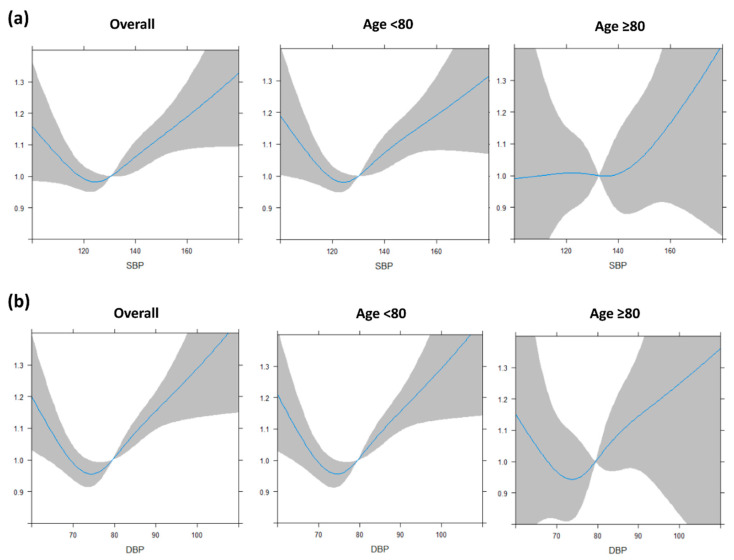
Systolic and diastolic blood pressure status of repeated measurement and risk of atrial fibrillation among elderly populations: (**a**) systolic blood pressure and (**b**) diastolic blood pressure. SBP, systolic blood pressure; DBP, diastolic blood pressure. The blue line shows relationship between hazard ratio of new-onset AF and blood pressure, and the gray area indicates the degree of confidence.

During 6.4 ± 2.1 years of follow-up and a total of 768,314 person-years, 4393, 3946, and 447 incident AF cases occurred in the overall, non-octogenarian, and octogenarian populations, respectively. The spline curves of the SBP and DBP and risk of AF in different age groups are presented in Figure 2. A U-shaped relationship between SBP or DBP and risk of AF was evident; however, the U-shaped relationship for SBP was not observed in the octogenarian population. There is the larger uncertainty in the older group because it is numerically small. The optimal SBP level associated with the lowest the risks of AF was 120–129 mm Hg in the overall population and the non-octogenarian population. The optimal DBP level with the lowest risk of AF was 70–79 mm Hg.

**Figure 3:**
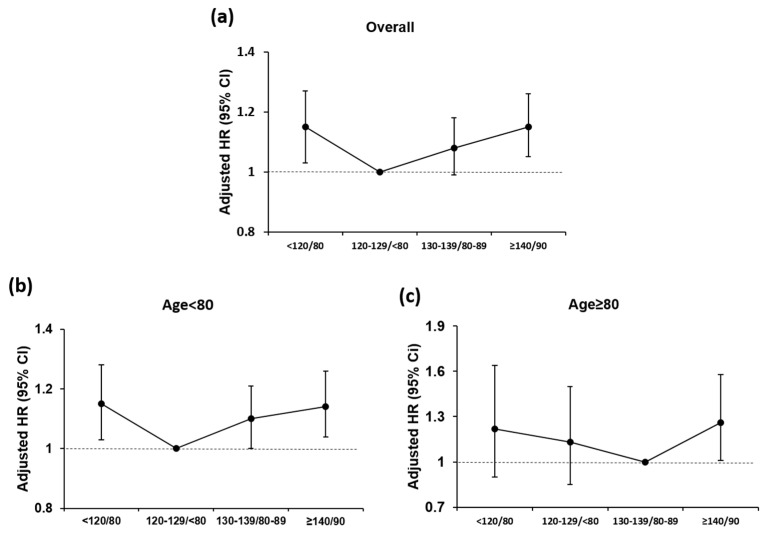
Blood pressure status of repeated measurement and risk of atrial fibrillation among elderly populations: (**a**) overall population, (**b**) age < 80 years, and (**c**) age ≥ 80 years. HR, hazard ratio.

After multivariable adjustment for potentially confounding clinical covariates, in non-octogenarians, the risk of AF was higher in patients with BP levels of <120/<80 and ≥140/90 mm Hg with adjusted hazard ratios (HR) of 1.15 (95% CI, 1.03–1.28, *p* < 0.001) and 1.14 (95% CI, 1.04–1.26, *p* < 0.001), respectively, compared to BP levels of 120–129/<80 mm Hg. Amongst octogenarians, the risk of AF was significantly higher in patients with BP levels of ≥140/90 mm Hg with an HR of 1.26 (95% CI, 1.01–1.58, *p* < 0.001) compared with the optimal BP level (130–139/80–90 mm Hg; Table 2, Figure 3).

### 3.3. BP and Incident AF in Patients with Treated Hypertension

**Figure 4:**
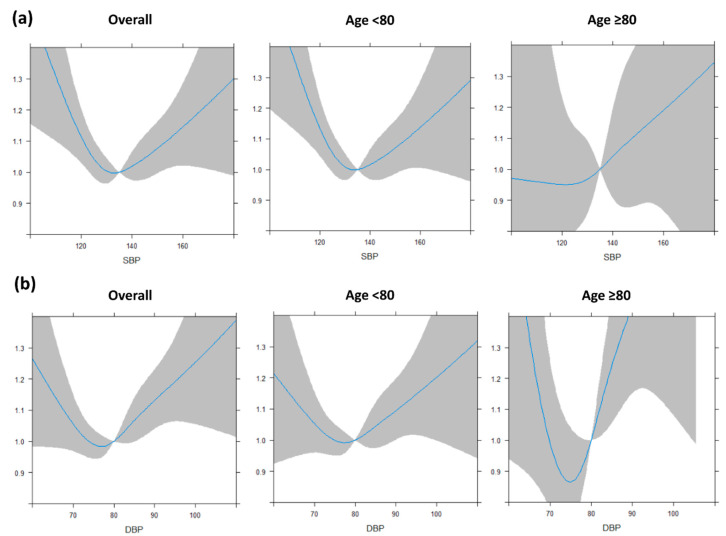
Systolic and diastolic blood pressure status of repeated measurement and risk of atrial fibrillation among elderly populations with antihypertensive medications: (**a**) systolic blood pressure and (**b**) diastolic blood pressure. SBP, systolic blood pressure; DBP, diastolic blood pressure. The blue line shows relationship between hazard ratio of new-onset AF and blood pressure, and the gray area indicates the degree of confidence.

With a total of 298,087 person-years follow-up of patients with treated hypertension, there were 2069, 1846, and 3636 incident AF cases occurring in the overall, non-octogenarian, and octogenarian populations, respectively. The spline curves of the SBP and DBP and risk of AF in different age groups are presented in Figure 4 and had a similar pattern as the spline curve for the general populations (Figure 2).

**Figure 5:**
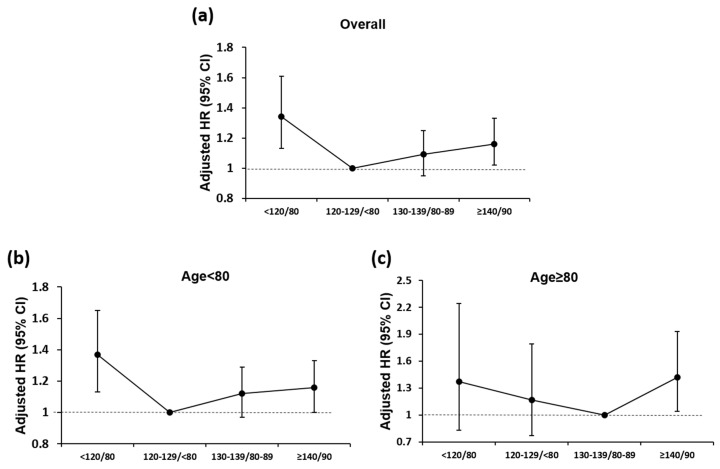
Blood pressure status of repeated measurement and risk of atrial fibrillation among elderly populations with antihypertensive medications: (**a**) overall population, (**b**) age < 80 years, and (**c**) age ≥ 80 years.

After multivariable adjustment for potentially confounding clinical covariates in the non-octogenarian population, the risk of AF was higher in patients with intensive BP control (<120/<80 mm Hg) and poor (≥140/90 mm Hg) BP control with adjusted HRs of 1.37 (95% CI, 1.13–1.65, *p* < 0.001) and 1.16 (95% CI, 1.0–1.33, *p* < 0.001), respectively, compared to those with optimal BP control (120–129/<80 mm Hg). In octogenarians, the risk of AF was significantly higher in patients with a BP levels of ≥140/90 mm Hg with an HR of 1.42 (95% CI, 1.04–1.93, *p* <0.001) compared to those with optimal BP level (130–139/80–90 mm Hg; Table 3, Figure 5).

### 3.4. Serious Adverse Events according to BP Status in Different Age Groups

The incidence rate and HR of serious adverse events according to BP status in patients with hypertension treatment are presented in Table 4. In octogenarians, patients with intensive BP control (<120/<80 mm Hg) showed more hypotension requiring hospitalization than those with a BP levels of 130–139/80–90 mm Hg with an adjusted HR of 2.06 (95% CI, 1.12–3.81, *p* < 0.001). The composite adverse events (including hypotension requiring hospitalization, syncope, bradycardia, electrolyte abnormality, injurious falls, and acute kidney injury) were numerically more frequent but non-statistically significant in patients with intensive and optimal BP control compared to those with a BP level of 130–139/80–89 mm Hg.

## 4. Discussion

In this large nationwide study on the impact of hypertension on incident AF, our principal findings are that BP levels of 120–129/<80 mm Hg were associated with a lower risk of incident AF in non-octogenarians. Amongst octogenarians, an average 10 mm Hg higher BP level of 130–139/80–89 mm Hg was more optimal to prevent AF. Second, octogenarians with intensive BP control (<120/<80 mm Hg) showed more hypotension requiring hospitalization compared with BP level of 130–139/80–90 mm Hg. Hence, a less strict BP level may be better to prevent AF and adverse effects amongst octogenarians.

The relationship between high BP and high incidence of AF supports the importance of BP control to prevent AF in patients with hypertension. Hypertension is the most common and important modifiable AF risk factor [2,3,22,23]. In a SPRINT sub-analysis, intensive BP control targeting SBP < 120 mm Hg was related with a lower risk of new-onset AF [13]. In contrast, the present study found a U-shaped relationship was observed between BP and incident AF in both non-octogenarians and octogenarians. This U-shaped relationship could be related to several unique aspects of our study cohort.

The present study recruited participants aged over 60 years, with the median age of our population being 71.7 years, much older than previous studies [11,12,13], and 6.8% of overall population were individuals of age over 80 years. The elderly population had more comorbidities, and these factors may influence the U-shaped relationship. Since the incidence of AF increases with age, we evaluated the relationship between the optimal BP and AF risk for BP management in older individuals. When comparing the relationship between AF and DBP, a U-shaped pattern was also observed in the overall population and patients with hypertension.

### 4.1. Optimal BP Levels and Incident AF in Octogenarians

Amongst octogenarians, an average 10 mm Hg higher BP level of 130–139/80–89 mm Hg was more optimal to prevent AF; this compares to non-octogenarians where the optimal BP level was 120–129/<80 mm Hg. However, the management of hypertension in octogenarians offers more challenges than in non-octogenarians. Elderly patients (age > 80 years) have more comorbidities and higher risks other organ damage than patients aged under 80 years. In old patients, physicians should consider the risks and benefits when controlling BP due to aggravation of postural hypotension and reduction of renal function [16]. Also, intensive BP control has been related to increased serious adverse events such as hypotension, syncope, electrolyte abnormalities, and acute kidney injury [14]. Our results show an increased risk of hypotension requiring hospitalization in the intensive BP control group (BP < 120/80 mm Hg) compared with BPs 130–139/80–89 mm Hg. Even though other adverse events did not show significant differences, the composite outcome of adverse events showed a trend towards an increased risk in patients with intensive control (BP < 120/80 mm Hg) and optimal BP control (120–129/<80 mm Hg) compared to patients with BP levels of 130–139/80–89 mm Hg.

### 4.2. Limitations

The study has several limitations. First, in such studies using administrative databases, coding inaccuracies can lead to errors. Hence, we applied the definition that we had already validated in previous studies to minimize the problem [21,24,25,26,27,28]. Second, since the health examination of individuals was conducted in different hospitals and clinics, a uniformity of BP measurement could not be achieved. Third, the arbitrary cut-offs across continuous distributions (e.g., age, BP) were used to compare groups using simple binary statistical tests in this study. While the simplification can illustrate possible trends, it does automatically lead to a loss of detail in data analysis. Fourth, those who survive into their ninth decade are already a positively selected group and presumably with useful healthy characteristics. Fifth, since data about the types of AF and differential diagnosis between AF and atrial flutter (AFL) were not available, we could not investigate about the difference of optimal BP level according to the types of AF or AFL. Finally, hypertension and AF are associated with renal dysfunction. In our study, CKD was defined using the medical record with ICD-10 codes. There was no data on proteinuria. The lack of data on proteinuria, which is one of the criteria for CKD, may lead to low accuracy in defining CKD. This is one of the limitations of the study. Despite these limitations, the study is the first assessment to investigate the association between BP levels and incidental AF in a nationwide elderly population.

## 5. Conclusions

A U-shaped relationship for the development of incident AF was evident in non-octogenarians, and BP levels of 120–130/<80 mm Hg were associated the lowest risk of incident AF. Compared to non-octogenarians, the lowest risk of AF was associated with higher BP levels of 130–139/80–89 mm Hg amongst octogenarians.

## Figures and Tables

**Table 1:** Comparison of clinical characteristics between the non-octogenarian and the octogenarian populations.

	All Population (*N* = 115,866)	Age		
		Age < 80 (*n* = 108,035)	Age ≥ 80 (*n* = 7831)	*p*-Value
Age, years	71.7 (69.5–74.6)	71.2 (69.3–74.0)	82.2 (81.0–84.5)	<0.001
Male	53,609 (46.3)	50,391 (46.6)	3218 (41.1)	<0.001
Systolic BP	130.5 (122.5–140.0)	130.0 (122.5–140.0)	132.5 (124.5–142.5)	<0.001
Diastolic BP *	79.5 (74.0–85.0)	79.5 (74.0–85.0)	79.5 (73.5–85.0)	0.017
Economic state *	7.0 (4.0–9.0)	7.0 (4.0–9.0)	7.0 (3.0–9.0)	0.001
Alcohol				<0.001
No drinking	64,681 (80.9)	60,075 (80.6)	4606 (85.8)	
Moderate drinking	5982 (7.5)	5673 (7.6)	309 (5.8)	
Heavy drinking †	9243 (11.6)	8789 (11.8)	454 (8.5)	
Smoking				<0.001
Non-smoker or quit ≥12 months	63,389 (79.3)	58,993 (79.1)	4396 (81.9)	
Quit <12 months	5732 (7.2)	5351 (7.2)	381 (7.1)	
Current smoker	10,785 (13.5)	10,193 (13.7)	592 (11.0)	
Comorbidities				
Hypertension	46,519 (40.1)	42,883 (39.7)	3636 (46.4)	<0.001
Diabetes	14,767 (12.7)	13,881 (12.8)	886 (11.3)	<0.001
Dyslipidemia	34,200 (29.5)	32,369 (30.0)	1831 (23.4)	<0.001
Chronic kidney disease	997 (0.9)	913 (0.8)	84 (1.1)	0.041
Anemia	17,715 (15.3)	15,715 (14.6)	2000 (25.6)	<0.001
Hyperthyroidism	2462 (2.1)	2341 (2.2)	121 (1.5)	<0.001
Hypothyroidism	2725 (2.4)	2583 (2.4)	142 (1.8)	0.001
COPD	6960 (6.0)	6262 (5.8)	698 (8.9)	<0.001
Liver disease	23,559 (20.3)	22,335 (20.7)	1224 (15.6)	<0.001
HCMP	156 (0.1)	149 (0.1)	7 (0.1)	0.331
Osteoporosis	33,139 (28.6)	30,569 (28.3)	2570 (32.8)	<0.001
Medications				
Aspirin	19,315 (16.7)	17,957 (16.6)	1358 (17.3)	0.102
P2Y12 inhibitor	872 (0.8)	816 (0.8)	56 (0.7)	0.742
ACE-inhibitor/ARB	18,425 (15.9)	17,110 (15.8)	1315 (16.8)	0.027
Beta blocker	18,115 (15.6)	16,815 (15.6)	1300 (16.6)	0.015
Calcium channel blocker	30,370 (26.2)	27,945 (25.9)	2425 (31.0)	<0.001
Statin	11,918 (10.3)	11,333 (10.5)	585 (7.5)	<0.001
Diuretics	23,665 (20.4)	21,728 (20.1)	1937 (24.7)	<0.001
MRA	1677 (1.4)	1533 (1.4)	144 (1.8)	0.003

BP, blood pressure; COPD, chronic obstructive pulmonary disease; HCMP, hypertrophic cardiomyopathy; ACE, angiotensin converting enzyme; ARB, angiotensin II receptor blocker; MRA, mineralocorticoid receptor antagonist. * Several parameters including diastolic BP, economic state showed exactly the same median values. However, still significant (admittedly *p* < 0.05) statistical differences because significantly different quartile values. † Male: >112 g/week or >42 g/day, Female: >56 g/week or >28 g/day. (14g per a glass). Values are presented as median (Q1-Q3 quartiles (25th and 75th percentiles)) or %.

**Table 2:** Incidence Rate of Atrial Fibrillation According to Blood Pressure.

	All Population			Age < 80 Years			Age ≥ 80 Years		
Group	No. /Total No. 4393 /115,866	Incidence Rate per 1000 Person-Years (95 CI)	Hazard Ratio (95 CI)	No. /Total No. 3946 /108,035	Incidence Rate Per 1000 Person-Years (95 CI)	Hazard Ratio (95 CI)	No. /Total No. 447 /7831	Incidence Rate Per 1000 Person-Years (95 CI)	Hazard Ratio (95 CI)
**<120** **/<80**	713 /19,712	5.47 (5.07–5.88)	1.15 (1.03–1.27)	649 /18,556	5.24 (4.84–5.66)	1.15 (1.03–1.28)	64 /1156	9.78 (7.53–12.49)	1.22 (0.90–1.64)
**120–129** **/<80**	721 /21,463	5.13 (4.76–5.52)	1 (reference)	647 /20,104	4.88 (4.51–5.27)	1 (reference)	74 /1359	9.37 (7.35–11.76)	1.13 (0.85–1.50)
**130–139** **/80–89**	1526 /40,667	5.67 (5.39–5.96)	1.08 (0.99–1.18)	1386 /37,949	5.47 (5.19–5.77)	1.10 (1.00–1.21)	140 /2718	8.65 (7.28–10.21)	1 (reference)
**≥140** **/≥90**	1433 /34,024	6.28 (5.96–6.62)	1.15 (1.05–1.26)	1264 /31,426	5.95 (5.63–6.29)	1.14 (1.04–1.26)	169 /2598	10.76 (9.20–12.52)	1.26 (1.01–1.58)

CI, confidence interval.

**Table 3:** Incidence Rate of Atrial Fibrillation According to Blood Pressure in Patients with Treated Hypertension.

	All Population			Age <80 Years			Age ≥ 80 Years		
Group	No./ Total No. 2069 /46,519	Incidence Rate per 1000 Person-Years (95 CI)	Hazard Ratio (95 CI)	No./ Total No. 1846 /42,883	Incidence Rate per 1000 Person-Years (95 CI)	Hazard Ratio (95 CI)	No./ Total No. 223 /3636	Incidence Rate Per 1000 Person-Years (95 CI)	Hazard Ratio (95 CI)
**Intensive control** **(<120/80)**	210 /4159	8.22 (7.14–9.41)	1.34 (1.13–1.61)	189 /3814	7.96 (6.87–9.18)	1.37 (1.13–1.65)	21 /345	11.56 (7.16–17.67)	1.37 (0.83–2.24)
**Optimal control** **(120–129** **/<80)**	290 /7466	6.22 (5.52–6.97)	1 (reference)	257 /6893	5.91 (5.21–6.68)	1 (reference)	33 /573	10.36 (7.13–14.55)	1.17 (0.77–1.79)
**Suboptimal control** **(130–139** **/80–89)**	735 /17,268	6.66 (6.19–7.16)	1.09 (0.95–1.25)	667 /15,934	6.49 (6.01–7.01)	1.12 (0.97–1.29)	68 /1334	8.90 (6.91–11.29)	1 (reference)
**Poor control** **(≥140/90)**	834 /17,626	7.22 (6.74–7.73)	1.16 (1.02–1.33)	733 /16,242	6.83 (6.34–7.34)	1.16 (1.00–1.33)	101 /1384	12.46 (10.15–15.14)	1.42 (1.04–1.93)

CI, confidence interval.

**Table 4:** Incidence Rate and Hazard Ratio of Serious Adverse Events According to Blood Pressure Status in Patients with Hypertension Treatment.

		Blood Pressure Status			
		<120/80 mmHg	120–129/<80 mmHg	130–139/80–89 mmHg	≥140/90 mmHg
***Overall***	No. of total	4159	7466	17,268	17,626
**Composite event**	No. of events, HR (95 CI)	341 1.08 (0.94–1.23)	580 1 (reference)	1269 0.95 (0.86–1.04)	1437 1.01 (0.92–1.11)
**Hypotension requiring hospitalization**	No. of events, HR (95 CI)	95, 1.15 (0.89–1.48)	151, 1 (reference)	348, 0.99 (0.82–1.20)	366, 0.98 (0.81–1.19)
**Syncope**	No. of events HR (95 CI)	33, 1.08 (0.70–1.67)	55, 1 (reference)	155, 1.23 (0.90–1.67)	157, 1.18 (0.86–1.60)
**Bradycardia**	No. of events HR (95 CI)	22 0.99 (0.59–1.67)	41 1 (reference)	96 1.01 (0.70–1.45)	97 0.96 (0.67–1.39)
**Electrolyte abnormality**	No. of events HR (95 CI)	142 0.99 (0.80–1.21)	263 1 (reference)	523 0.86 (0.75–1.00)	646 1.01 (0.88–1.17)
**Injurious falls**	No. of events HR (95 CI)	11 1.11 (0.52–2.37)	17 1 (reference)	46 1.18 (0.67–2.06)	41 0.97 (0.55–1.71)
**Acute kidney injury**	No. of events HR (95 CI)	106 1.18 (0.93–1.50)	172 1 (reference)	364 0.90 (0.75–1.08)	433 0.96 (0.80–1.14)
***Age < 80 Years***	No. of total	3814	6893	15,934	16,242
**Composite event**	No. of events HR (95 CI)	298 1.08 (0.94–1.25)	506 1 (reference)	1124 0.96 (0.87–1.07)	1279 1.03 (0.93–1.15)
**Hypotension requiring hospitalization**	No. of events HR (95 CI)	80 1.13 (0.85–1.49)	129 1 (reference)	313 1.05 (0.85–1.28)	327 1.03 (0.84–1.27)
**Syncope**	No. of events HR (95 CI)	31 1.28 (0.81–2.02)	44 1 (reference)	141 1.39 (0.99–1.96)	143 1.33 (0.95–1.87)
**Bradycardia**	No. of events HR (95 CI)	18 0.88 (0.50–1.55)	38 1 (reference)	91 1.03 (0.71–1.51)	87 0.9. (0.63–1.36)
**Electrolyte abnormality**	No. of events HR (95 CI)	122 0.98 (0.79–1.22)	228 1 (reference)	453 0.87 (0.74–1.02)	573 1.04 (0.8–1.22)
**Injurious falls**	No. of events HR (95 CI)	10 1.02 (0.45–2.24)	17 1 (reference)	40 1.04 (0.59–1.84)	38 0.91 (0.51–1.61)
**Acute kidney injury**	No. of events HR (95 CI)	96 1.25 (0.96–1.61)	148 1 (reference)	317 0.91 (0.75–1.11)	378 0.98 (0.81–1.18)
**Age ≥ 80 Years**	No of total	345	573	1334	1384
**Composite event**	No. of events HR (95 CI)	43 1.32 (0.94–1.86)	74 1.25 (0.94–1.66)	145 1 (reference)	158 1.03 (0.82–1.29)
**Hypotension requiring hospitalization**	No. of events HR (95 CI)	15 2.06 (1.12–3.81)	22 1.64 (0.96–2.81)	35 1 (reference)	39 1.08 (0.68–1.72)
**Syncope**	No. of events HR (95 CI)	2 0.57 (0.13–2.51)	11 1.69 (0.74–3.85)	14 1 (reference)	14 1.69 (0.74–3.85)
**Bradycardia**	No. of events HR (95 CI)	4 3.73 (0.97–14.32)	3 0.98 (0.19–5.14)	5 1 (reference)	10 2.02 (0.68–6.01)
**Electrolyte abnormality**	No. of events HR (95 CI)	20 1.21 (0.73–1.99)	35 1.18 (0.78–1.79)	70 1 (reference)	73 0.98 (0.70–1.36)
**Injurious falls**	No. of events HR (95 CI)	1 0.75 (0.09–5.90)	0	6 1 (reference)	3 0.44 (0.11–1.72)
**Acute kidney Injury**	No. of events HR (95 CI)	10 1.06 (0.53–2.11)	24 1.27 (0.77–2.11)	47 1 (reference)	55 1.02 (0.69–1.52)

CI, confidence interval.

## Supplementary Materials

The following are available online at https://www.mdpi.com/2077-0383/9/9/2988/s1; Supplementary Table S1: Definitions and International Classification of Disease-10th Revision (ICD-10) codes used for defining the comorbidities and clinical outcomes; Supplementary Table S2: Comparison of baseline characteristics in patients with different blood pressure levels in overall population.
