# Supplementary material for: What Is the Ideal Blood Pressure Threshold for the Prevention of Atrial Fibrillation in Elderly General Population?
Source: J Clin Med. 2020 Sep 16;9(9):2988. doi: 10.3390/jcm9092988 (PMC7563734; doi:10.3390/jcm9092988)
Supplement: Supplementary file 1 [file jcm-09-02988-s001.pdf]

Supplementary Table S1. Definitions and International Classification of Disease-10th Revision (ICD-10) codes used for defining the comorbidities and clinical outcomes

	Definitions	ICD-10 Codes or Conditions
Comorbidities		
Atrial fibrillation	Defined from diagnosis*	ICD-10: I48
Heart failure	Defined from diagnosis*	ICD-10: I11.0, I50, I97.1
Hypertension	Defined from diagnosis*	ICD-10: I10, I11, I12, I13, I15 and antihypertensive medication
Diabetes mellitus	Defined from diagnosis* plus treatment	ICD-10: E10, E11, E12, E13, E14<br>Treatment: all kinds of oral antidiabetics and insulin.
Dyslipidemia	Defined from diagnosis*	ICD-10: E78
Ischemic stroke	Defined from diagnosis*	ICD-10: I63, I64
Transient ischemic attack	Defined from diagnosis*	ICD-10: G45
Hemorrhagic stroke	Defined from diagnosis*	ICD-10: I60, I61, I62
Myocardial infarction	Defined from diagnosis*	ICD-10: I21, I22, I25.2
Peripheral arterial disease	Defined from diagnosis*	ICD-10: I70.0, I70.1, I70.2, I70.8, I70.9
Chronic kidney disease	Defined from eGFR or diagnosis*<br>(if laboratory value was not available, diagnosis code was used)	eGFR <60mL/min per 1.73 m^2<br>ICD-10: N18, N19
End-stage renal disease	Defined from national registry for severe illness.	Patients with end-stage renal disease undergoing chronic dialysis or received a kidney transplant.
Hypertrophic cardiomyopathy	Defined from at least one records of either inpatient or outpatient diagnoses	ICD-10: I42.1, I42.2
Sleep apnea	Defined from diagnosis*	ICD-10: G47.3
Proteinuria	Defined from laboratory data (if laboratory value was not available, diagnosis code was used)	Urine dipstick proteinuria 1+ or higher (ICD-10: N06, N391, N392, R80)
Osteoporosis	Defined from diagnosis*	ICD-10: M80, M81, M82 (except M82.0)
Hyperthyroidism	Defined from diagnosis*	ICD-10: E05
Hypothyroidism	Defined from diagnosis*	ICD-10: E03
Chronic Liver disease	Defined from diagnosis of chronic liver disease, cirrhosis, and hepatitis	ICD-10: B18, K70, K71, K72, K73, K74, K76.1
Chronic obstructive pulmonary disease	Defined from diagnosis* plus treatment	ICD-10: J42, J43(except J43.0), J44<br>Treatment: SABA, SAMA, LABA, LAMA, ICS, ICS+LABA, or methylxanthine (>1 months).
Malignancy	Defined from diagnoses of cancer (non-benign)	ICD-10: C00-C97
Clinical outcomes		

Ischemic stroke	Defined from any discharge diagnoses with concomitant imaging studies	ICD-10: I63, I64
Systemic embolism	Defined from admission diagnosis or related death	ICD-10: I74, N280 (including renal infarction)
Heart failure admission	Defined from admission diagnosis (including only main and first sub-diagnosis)	ICD-10: I11.0, I50, I97.1

* To ensure accuracy, comorbidities were established based on one inpatient or two outpatient records of ICD-10 codes in the database. eGFR, estimated glomerular filtration rate; ICD-10, International Classification of Diseases-10th Revision.

Supplementary Table S2. Comparison of baseline Characteristics in patients with different blood pressure levels in overall population.

	**Non-octogenarian (108,035)**					**Octogenarian (*n* = 7831)**				
	**<120/80 mmHg (*n* = 18,556)**	**120-129 /<80mmHg (*n* = 20,104)**	**130-139 /80-89 mmHg (*n* = 37,949)**	**≥140/90 mmHg (*n* = 31,426)**	***p* value**	**<120/80 mmHg (*n* = 1156)**	**120-129 /<80mmHg (*n* = 1359)**	**130-139 /80-89 mmHg (*n* = 2718)**	**≥140/90 mmHg (*n* = 2598)**	***p* value**
Age, years	70.5 [68.8-73.7]	71.2 [69.4-74.0]	71.2 [69.3-73.9]	71.5 [69.4-74.1]	<0.001	82.5 [81.4-84.7]	82.2 [80.7-84.5]	82.2 [80.9-84.4]	82.2 [81.1-84.6]	0.047
Male	8446 (45.5)	9146 (45.5)	17865 (47.1)	14934 (47.5)	<0.001	501 (43.3)	576 (42.4)	1109 (40.8)	1032 (39.7)	0.141
Systolic BP	114.0 [110.0-116.5]	124.5 [121.5-126.5]	132.5 [129.5-135.0]	145.5 [141.5-153.0]	<0.001	114.5 [110.0-116.5]	125.0 [121.5-126.5]	132.5 [130.0-135.0]	147.5 [142.5-155.0]	<0.001
Diastolic BP	70.0 [65.5-74.5]	75.0 [71.0-76.5]	80.0 [78.0-84.0]	87.0 [82.0-91.0]	<0.001	70.0 [65.0-74.0]	74.5 [70.0-75.0]	80.0 [76.5-83.0]	85.0 [80.0-90.0]	<0.001
Economic state	7.0 [4.0-9.0]	7.0 [4.0-9.0]	7.0 [4.0-9.0]	7.0 [3.0-9.0]	<0.001	7.0 [3.0-9.0]	7.0 [4.0-9.0]	7.0 [3.0-9.0]	7.0 [3.0-9.0]	
Alcohol					<0.001					0.414
No drinking	11,287 (76.6)	11,500 (82.0)	20,844 (80.0)	16,444 (78.0)		683 (86.1)	793 (86.5)	1612 (85.2)	1518 (85.9)	
Moderate drinking	927 (6.9)	1008 (7.2)	2008 (7.7)	1730 (8.2)		49 (6.2)	47 (5.1)	101 (5.3)	112 (6.3)	
Heavy drinking	1164 (8.7)	1508 (10.8)	3209 (12.3)	2908 (13.8)		61 (7.7)	77 (8.4)	178 (9.4)	138 (7.8)	
Smoking					<0.001					0.062
Non-smoker or quit ≥12 months	10,242 (76.6)	11,026 (78.7)	20,678 (79.3)	17,047 (80.9)		632 (79.7)	745 (81.2)	1557 (82.3)	1462 (82.7)	

Quit <12 months	894 (6.7)	1,018 (7.3)	1,966 (7.5)	1,473 (7.0)		52 (6.6)	62 (6.8)	130 (6.9)	137 (7.7)	
Current smoker	2242 (16.8)	1972 (14.1)	3417 (13.1)	2562 (12.2)		109 (13.7)	110 (12.0)	204 (10.8)	169 (9.6)	
**Comorbidities**										
Hypertension	3814 (20.6)	6893 (34.3)	15,934 (42.0)	16,242 (51.7)	<0.001	345 (29.8)	573 (42.2)	1334 (49.1)	1384 (53.3)	<0.001
Diabetes	1936 (10.4)	2687 (13.4)	4920 (13.0)	4338 (13.8)	<0.001	126 (10.9)	174 (12.8)	328 (12.1)	258 (9.9)	0.021
Dyslipidemia	4940 (26.6)	6308 (31.4)	11,699 (30.8)	9422 (30.0)	<0.001	262 (22.7)	334 (24.6)	655 (24.1)	580 (22.3)	0.28
Chronic kidney disease	137 (0.7)	164 (0.8)	327 (0.9)	285 (0.9)	0.232	14 (1.2)	12 (0.9)	33 (1.2)	25 (1.0)	0.687
Anemia	3308 (17.8)	3197 (15.9)	5250 (13.8)	3960 (12.6)	<0.001	344 (29.8)	379 (27.9)	691 (25.5)	586 (22.6)	<0.001
Hyperthyroidism	406 (2.2)	527 (2.6)	798 (2.1)	610 (1.9)	<0.001	11 (1.0)	20 (1.5)	53 (1.9)	37 (1.4)	0.116
Hypothyroidism	506 (2.7)	576 (2.9)	860 (2.3)	641 (2.0)	<0.001	18 (1.6)	26 (1.9)	49 (1.8)	49 (1.9)	0.901
COPD	1172 (6.3)	1212 (6.0)	2239 (5.9)	1639 (5.2)	<0.001	124 (10.7)	136 (10.0)	256 (9.4)	182 (7.0)	<0.001
Liver disease	3856 (20.8)	4328 (21.5)	8000 (21.1)	6151 (19.6)	<0.001	198 (17.1)	233 (17.1)	442 (16.3)	351 (13.5)	0.003
HCMP	18 (0.1)	28 (0.1)	53 (0.1)	50 (0.2)	0.349	1 (0.1)	5 (0.4)	1 (0.0)	0 (0.0)	0.002
Osteoporosis	5612 (3.02)	6008 (29.9)	10,728 (28.3)	8221 (26.2)	<0.001	367 (31.7)	462 (34.0)	941 (34.6)	800 (30.8)	0.016
Medications										
Aspirin	1970 (10.6)	3068 (15.3)	6514 (17.2)	6405 (20.4)	<0.001	147 (12.7)	224 (16.5)	500 (18.4)	487 (18.7)	<0.001
P2Y12 inhibitor	126 (0.7)	163 (0.8)	290 (0.8)	237 (0.8)	0.511	8 (0.7)	10 (0.7)	18 (0.7)	20 (0.8)	0.972
ACE-inhibitor /ARB	1579 (8.5)	2803 (13.9)	6245 (16.5)	6483 (20.6)	<0.001	112 (9.7)	217 (16.0)	489 (18.0)	497 (19.1)	<0.001
Beta blocker	1,413 (7.6)	2,621 (13.0)	6,121 (16.1)	6,660 (21.2)	<0.001	116 (10.6)	187 (13.8)	479 (17.6)	518 (19.9)	<0.001
Calcium channel blocker	2227 (12.0)	4456 (22.2)	10,510 (27.7)	10,752 (34.2)	<0.001	224 (19.4)	398 (29.3)	886 (32.6)	917 (35.3)	<0.001
Statin	1613 (8.7)	2230 (11.1)	4089 (10.8)	3401 (10.8)	<0.001	75 (6.5)	110 (8.1)	201 (7.4)	199 (7.7)	0.469
Diuretics	1946 (10.5)	3474 (17.3)	8084 (21.3)	8224 (26.2)	<0.001	187 (16.2)	298 (21.9)	726 (26.7)	726 (27.9)	<0.001

Values are presented as median (Q1-Q3 quartiles [25th and 75th percentiles]) or %. ACE, angiotensin converting enzyme; AF, atrial fibrillation; ARB, angiotensin II receptor blocker; BP, blood pressure; COPD, chronic obstructive pulmonary disease; HCMP, hypertrophic cardiomyopathy.
